# A Social Psychological Model of Scientific Practices: Explaining Research Practices and Outlining the Potential for Successful Reforms

**DOI:** 10.5334/pb.496

**Published:** 2019-09-12

**Authors:** Lee Jussim, Jon A. Krosnick, Sean T. Stevens, Stephanie M. Anglin

**Affiliations:** 1 Rutgers University, US; 2 Stanford University, US; 3 New York University, US; 4 Hobart and William Smith Colleges, US

**Keywords:** Science reform, replication crisis, questionable research practices, meta-science

## Abstract

A crescendo of incidents have raised concerns about whether scientific practices in psychology may be suboptimal, sometimes leading to the publication, dissemination, and application of unreliable or misinterpreted findings. Psychology has been a leader in identifying possibly suboptimal practices and proposing reforms that might enhance the efficiency of the scientific process and the publication of robust evidence and interpretations. To help shape future efforts, this paper offers a model of the psychological and socio-structural forces and processes that may influence scientists’ practices. The model identifies practices targeted by interventions and reforms, and which practices remain unaddressed. The model also suggests directions for empirical research to assess how best to enhance the effectiveness of psychological inquiry.

## Introduction

During the last decade, psychologists have identified practices shown or hypothesized to yield irreplicable results and have implemented reforms thought to yield more robust evidence (Nelson, Simmons, & Simonsohn, 2018; Spellman, 2015). Much of this has unfolded without an overarching theory of the causes of scientists’ behavior that can guide implementation of innovations in scientific practice. This paper offers two related models. The first, a Social Psychological Model of Scientific Practices (the SPMSP), is an account of how institutional and individual forces may combine to direct the activities of scientists. It proposes mechanisms by which reforms may have constructive impact and highlights limitations to reforms intended to prevent suboptimal science. The second is a nearly identical model, differing from the SPMSP only in incorporating many of the practices and interventions that have emerged from the nascent science reform movement intended to improve the validity of psychological research (SPMSP-R). Both models can be used to generate empirically testable hypotheses about sources of (sub)optimal scientific practices.

The purpose of this paper is to take stock of and integrate much of the scholarship on reforming psychological science that has emerged relatively recently. It does not provide a bold new method, practice or statistic intended to solve some problem, and most of its ideas will be familiar to those engaged in science reform. Its main contributions, therefore, are to succinctly weave many of these ideas into an integrative and readable overview, which may be especially useful to scholars focused more on doing than reforming psychological (and possibly other) science(s), and to spur empirical research on improving psychological science.

## Suboptimal Practices in Psychology and Beyond: A Brief History

Psychologists have been ringing alarms about scientific practices for many decades but were mostly ignored until a confluence of events triggered the “replicability crisis” (Gelman, 2016; Spellman, 2015). Pivotal events in 2010–2012 included several high profile revelations of fraud (e.g., Funder, Levine, Mackie, Morf, Vazire, & West, 2014), and publication of research:

- Ostensibly demonstrating ESP (Bem, 2011) using methods and practices common in psychology, a finding many interpreted as more of an indictment of those methods and practices than as a demonstration of the reality of ESP (e.g., Gelman, 2016; Nelson, Simmons & Simonsohn, 2018; Spellman, 2015).
- Failing to replicate (Doyen, Klein, Pichon, & Cleeremans, 2012) a famous and massively cited study involving the behavioral effects of priming social stereotypes (Bargh, Chen, & Burrows, 1996).
- Documenting seemingly widespread use of questionable research practices, such as not reporting all dependent variables, conditions, or studies (John, Lowenstein & Prelec, 2012).
- Demonstrating how common practices yield illusory results (Simmons, Nelson, & Simonsohn, 2011).
- Showing that phenomena thought to be universal psychological realities did not always appear outside of Western college students (Heinrich, Heine & Norenzayan, 2010).
- Showing that researchers endorsed discriminating against other scientists, and scientific findings, based on politics (Inbar & Lammers, 2012).

In short, there was a flood tide of revelations of bad data, impossible conclusions, unreplicated studies, and dubious conclusions all built on suboptimal practices. Consequently, many psychologists became interested in implementing reforms with the goal of strengthening the replicability, validity, and credibility of psychological science (Nelson, Simmons, and Simonsohn, 2018; Nosek et al., 2012; Spellman, 2015). Next, therefore, we introduce a model providing an overview of the new understanding of what constitutes suboptimal practices, their likely sources, and what has been proposed by science reformers to improve psychological science.

## The Social Psychological Model of Scientific Practices

**Figure 1:**
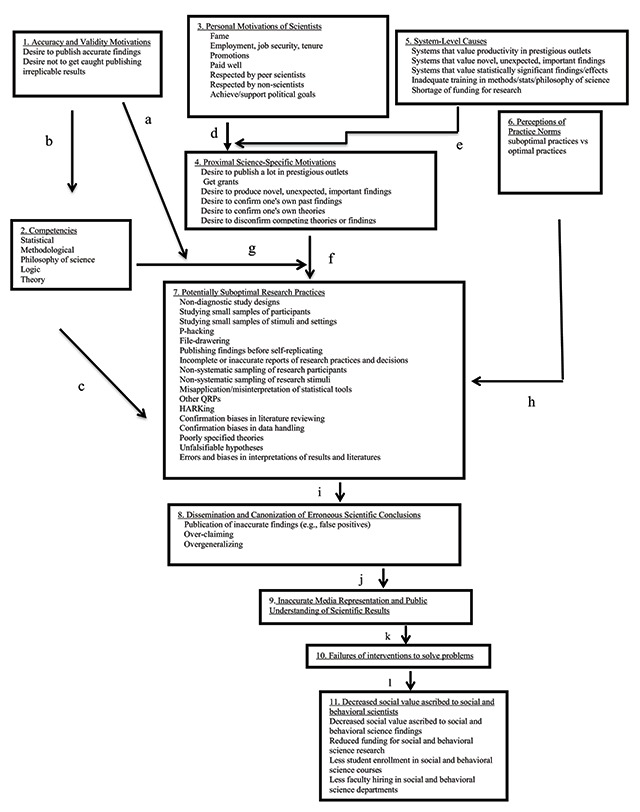
Social Psychological Model of Scientific Practices (SPMSP). Box 7 focuses on known problematic practices, but it should be obvious that, in each case, there is an opposite better practice (e.g., replace a nondiagnostic study design with a diagnostic one; replace small sample studies with large samples when possible).

Although the Social Psychological Model of Scientific Practices (SPMSP, Figure 1) is new, its ideas reflect the science reform literature. The model’s unique contribution is the integration of ideas and practices being widely discussed and implemented, making explicit presumed causal relations that have sometimes been implicit, and drawing connections between separate lines of scholarship. The SPMSP outlines both system-level and individual-level influences on scientific practices while assuming that individual scientists make decisions about which practices to implement. The SPMSP integrates many reform efforts in a single conceptual framework outlining the causes and consequences of scientific practices and mechanisms by which interventions might affect them.

The SPSPM is intended to capture a broad overview of much of what has been hypothesized as well as much that is, and is not, well-understood in the burgeoning science reform literature. As such, it is both descriptive and speculative. For example, it is descriptive in the sense that it includes scientists responding to institutional incentives; it is speculative because there is a great deal of doubt about how much changing incentives improves versus undermine the search for better scientific practices and more valid conclusions. Thus, most paths should be viewed as hypotheses; indeed, the value of each path in different contexts is an empirical question about which there is, in most cases, very little empirical evidence. Put differently, the SPSPM makes no assumption about either the weight or even sign of the different paths; indeed, the same path may be positive, negative, or zero in different contexts.

### Suboptimal Practices

The centerpiece of SPMSP is Box 7, suboptimal practices. Although the list of potentially suboptimal practices is nearly infinite, Box 7 highlights many of those most commonly identified in prior scholarship. These include methods (e.g., use of small samples of people, settings, or stimuli), statistics (p-hacking), practices (HARKing), or any of a variety of suboptimal theoretical or interpretive practices (for reviews, see, e.g., Fiedler, 2017; Fraley & Vazire, 2014; Gelman, 2016 Giner-Sorolla, 2012; John et al., 2012; Jussim, Crawford, Anglin, Stevens, & Duarte, 2016; Kerr, 1998; Spellman, 2015). Next, therefore, we discuss sources of these suboptimal practices, followed by a discussion of their consequences.

### Sources of (Sub)Optimal Practices

Suboptimal practices are the centerpiece of the SPMSP, because they increase the risk of producing invalid findings (Nelson et al., 2018; Simmons et al., 2011).^1^ The model proposes many possible causes of suboptimal practices. Therefore, we review each step of the model next.

#### Accuracy and validity motivations

Box 1 captures the idea that, often, perhaps most of the time, scientists seek truth and valid results. When scientists are primarily motivated by accuracy, the SPMSP proposes two routes through which suboptimal practices are minimized. The direct route (Path a) will likely often be negative because the more accuracy motivates scientists, the less they may use suboptimal practices, at least if they know what those practices are (see subsequent section on education).

There is also an indirect route: Paths b and c. Path b captures the idea that scientists high in accuracy/validity motivation will seek out the competencies (Box 2) needed to reduce suboptimal practices (Box 7) and produce valid research (e.g., methodological and statistical expertise, paying close attention to logical and philosophy of science issues, and to improved practices in science reform efforts). Although education per se is not shown in the model, a natural prediction would be that the more highly scientists are motivated by concerns about producing accurate and valid knowledge, the more likely they will be to obtain high levels of education about methods, practices, and statistics, for the purpose of producing better science.

Of course, education can also be seen as a certification process, and, therefore, something to be “gamed” in order to advance one’s career. For example, getting a Ph.D. at a prestigious institution could be intrinsically important because one believes one will learn best practices there ***or*** it could be extrinsically important and simply represent a useful stepping-stone towards advancing one’s career. Path c is expected to most typically be negative because, without relevant competencies scientists may use suboptimal practices out of ignorance. The SPMSP, therefore, predicts that competencies may mediate the effect of accuracy motivations on use of optimal (rather than suboptimal) practices.

#### Personal motivations and institutional incentives

We use the term “personal motivations” in Box 3 to capture the idea that scientists may be motivated by many things other than truth, including tenure, promotions, fame, and social approbation. Although all motivations can be viewed as “personal” in the sense that they are intra-psychic phenomena (i.e., a person holds them), the SPSPM distinguishes between motivations that can be viewed as advancing some sort of nonscientific interest (fame, fortune, policy) from those that involve seeking the truth. The point is not that these other goals are necessarily *antithetical* to truth (though sometimes they might be); it is, instead, that they are goals *other than* truth. Furthermore, in order to seek for truth, scientists need both a job and research funding sufficient to conduct research. As constructed, the existing system typically rewards number of publications, prestige of publication outlet, novelty of findings, and citation counts quite directly, and only indirectly, if at all, creating new knowledge by discovering things that are actually true. If even well-intentioned scientists must “play the game” in this manner, the validity of the science produced may suffer because they may seek to do that which will get them a job and funding, whether or not that involves seeking truth.

The common human motivations to succeed captured in Box 3 (Personal Motivations of scientists) then lead, via Path d, to a set of Proximal Science-Specific Motivations (Box 4) and which are highly incentivized by System-Level reward structures (Path e). Boxes 3, 4, and 5, and their connecting paths d and e indicate that these personal motivations may lead to many behaviors that in turn can lead to suboptimal practices. Path e reflects the idea that those incentives moderate the ways in which personal motivations lead scientists to seek goals other than truth – such as publishing in prestigious outlets, getting grants, producing highly cited papers promoting dramatic narratives, etc. ***if*** those goals are rewarded by institutions (as they usually are). Suboptimal practices are used, according to the SPMSP, in part, because they make it easier to publish and obtain grants, both of which are incentivized by institutions (Edwards & Roy, 2017; Giner-Sorolla, 2012; Nosek et al., 2012).

In a “publish or perish” system, scientists are incentivized to publish, *regardless of whether what they publish is valid or not*. Thus, scientists motivated by wanting to keep their jobs (e.g., tenure), receive raises and promotions, etc., are incentivized to publish, not to publish findings that are actually true. Although truth and publication are not necessarily at odds, nor are they necessarily aligned (Edwards & Roy, 2017; Nosek, Spies & Motyl, 2012). Although peer review is supposed to act as a quality control, in fact, reviewers are often seduced by compelling narratives (Jussim et al., 2016), and by implausibly “beautiful” results (Fraley & Vazire, 2014). Many other goals unrelated to accuracy (novelty, theory-confirming, personal aggrandizement, etc.) can lead (Path f) to suboptimal practices (Giner-Sorolla, 2012).

Furthermore, even though the SPSPM includes a path (c) whereby competence reduces suboptimal practices, it includes another (g) whereby it can have boomerang effects. People who are more educated or sophisticated sometimes engage in *more* confirmation bias, because they deploy their well-developed reasoning skills not to arrive at truths, but to defend their preferred beliefs (Kahan et al., 2012). For example, researchers with a vast array of statistical skills will have *even greater flexibility to* seek statistical significance than their less skilled colleagues, simply because they will be able to analyze the data in a greater variety of ways. Whether Path g is positive or negative, then, depends on whether increased scientific competencies will necessarily have the desired effect of reducing how much non-truth-seeking motivations (path f) produce suboptimal practices.

#### Norms

Box 6 refers to social norms that can influence scientific practices. It captures the idea that, like other people in other contexts, scientists often go along with what is normative in their discipline, whether or not the practice is (sub)optimal, and often taking for granted that it is optimal. Extensive research on conformity to social norms (Cialdini & Goldstein, 2004), understudied in science reform, raises the possibility that through normative or informational influence scientists may adopt particular scientific practices (Path h). Path h reflects the possibility that these practices could result from normative conformity (“everyone does it, so should I”) or informational conformity (“this must be the right way to do science”) to common suboptimal practices.

For example, psychological publications have routinely and unjustifiably reached broad and general conclusions on the basis of small and unrepresentative college student samples (Fraley & Vazire, 2014; Heinrich et al., 2010). However, doing so was so common for much of social psychology’s existence, that having done so is plausibly described as normative, until the science reform movement of 2011–12 came along raising major doubts (others may have raised doubts previously, but those doubts were largely ignored – see Gelman, 2016).

Similarly, despite American Psychological Association rules requiring otherwise, researchers have, historically, often not made their data or materials available to other scientists (Wicherts, Bakker, & Molenaar, 2011). Because there was no norm for open data, despite the rule requiring data availability, there were neither norms nor institutional/organizations practices that supported data sharing. Thus, about half of the requests for data once went unfulfilled (Wicherts et al., 2011).

Another norm has involved treating publication of statistically significant results as synonymous with establishing a scientific fact (Gelman, 2016). Of course, the recent history of failed replications (e.g., Finnigan & Corker, 2016; Open Science Collaboration, 2015; Yap et al., 2017) shows that, even when something is published, and based on a “statistically significant” finding, the finding may or may not be replicable or valid. What’s worse, even if the finding is valid, the conclusions or interpretations may not be (Jussim et al., 2016). Thus, a norm of treating a published finding as establishing a scientific fact constituted an egregious form of overconfidence, with two major real world consequences:

1. The psychological canon (claims widely believed by psychologists, and oft-repeated in textbooks and outlets of record) could become filled with claims and conclusions that turned out either to be false or, at best, not well-established (for examples involving person perception, see Jussim, 2012; for examples involving stereotype threat, see, e.g., Flore & Wichert, 2015; Stoets & Geary, 2012).
2. Those who raised questions about the validity of such pseudo-facts were often treated as if they were engaging in personal attacks on the original authors and were subject to insults and bullying plausibly described as attempts at silencing (Roberts, 2019).

## A Brief Detour: How Should Valid New Knowledge be Established in Psychology?

An implicit assumption underlying most of this review and model is that credible scientific knowledge in psychology is not established by any single paper, even one with a large sample or many internal replications. Evolutionary biologist Stephen Jay Gould (1981) defined scientific fact as follows: “In science, ‘fact’ can only mean ‘confirmed to such a degree that it would be perverse to withhold provisional assent.’” One implication of this definition is that when something is *not* so well established, it is not perverse to withhold provisional assent; thus, it may or may not be true or valid, but it is not an *established* scientific fact. Another is that some belief might lose its status as “fact” should new information come along showing that it was not really as well established as one once thought.

Indeed, even referring to conclusions derived from psychological research as “facts” may be viewed as overly reifying what is learned from scientific research. Thus, in the remainder of this article, we refer to knowledge, rather than facts. A major goal of scientific endeavor, then, is to discover and establish valid new knowledge.

A minimum of four conditions should be met to plausibly describe a new discovery in psychology as valid new knowledge. (all are necessary, none are sufficient individually):

1. Something must be found.
2. It must be subject to rigorous, pre-registered attempts at replication by researchers independent of the team providing the discovery.
3. Most of those rigorous pre-registered replication attempts must succeed.
4. Pre-registered meta-analyses of those pre-registered studies must reveal that the phenomenon exists even after attempts to assess and remove biases in the literature.

**Figure 2:**
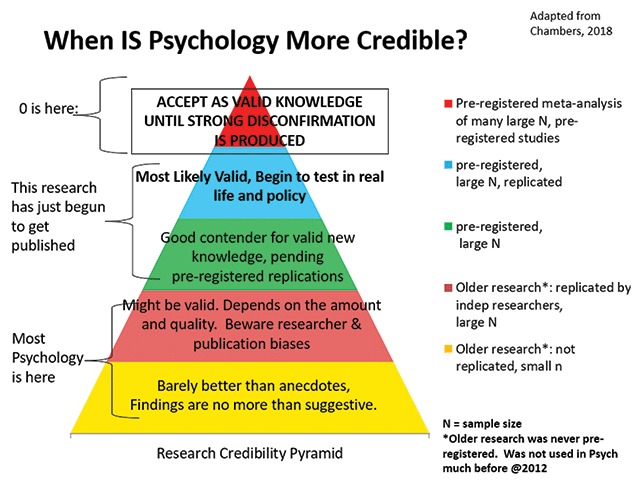
The Research Credibility Pyramid.

Figure 2 shows the Research Credibility Pyramid (RCP), which captures the view proposed here regarding when some findings in psychology are plausibly described as having established valid new knowledge. It treats much prior conventional research in psychology as preliminary more than definitive. In the absence of pre-registered replications by independent teams of researchers, the RCP does not consider any finding to be “valid knowledge” (it may be a “fact” that such and such a study was conducted and reported such and such a result, but that says nothing about the replicability or the validity of that result). Thus the conclusion cannot be treated as “valid new knowledge” until far more confirmation is obtained by independent teams. Inasmuch as pre-registration is a relatively recent development in psychology, and there have been no published pre-registered meta-analyses of exclusively pre-registered studies of which we are aware, the RCP strongly suggests that much prior work in psychology is best viewed with a skeptical eye.

The RCP is intended to identify standards sufficient to consider a finding a “valid new knowledge” in psychology. However, it may not always be applicable, especially to older researcher that pre-dates pre-registration. Certain basic phenomena are so well-established and widely established by older literatures that they are plausibly considered facts. For example, Stroop effects have been obtained so widely that they can be considered facts (MacLeod, 1991). The basic race IAT (implicit association test) effect, whereby most people find it easier to categorize target words into (variations on) Black/bad White/good versus Black/good White/bad (e.g., Lane, Banaji, Nosek, & Greenwald, 2007), has also been obtained so widely as to be plausibly considered a fact.

However, with respect to *understanding the nature of those facts*, we would add a fifth condition, one that is not reflected in the RCP:

5. The conclusions and interpretations regarding some phenomenon, even when its evidentiary basis is sufficiently strong to be considered “valid new knowledge” needs to be subjected to long and intense skeptical scrutiny by the scientific community.

We may know that Stroop or IAT effects occur, and yet there can still be a great deal of uncertainty about what they mean or how to interpret them (Fiedler, Messner, & Bluemke, 2006; MacLeod, 1991). Condition 5 is not shown in the RCP, because the RCP focuses exclusively on establishing whether some phenomenon is established as occurring; it does not address how or when an understanding of that phenomenon also deserves to be treated as valid new knowledge. If such “understandings” can be translated into testable empirical hypotheses about processes, the “knowledge-establishing” process begins anew at the base of the pyramid. If they cannot be so translated, it becomes scientifically difficult or impossible to distinguish among competing interpretations.

## Downstream Consequences of Suboptimal Practices

Boxes 8 through 11 of Figure 1 identify some of the consequences suboptimal practices. Box 8 is short and sweet because the bottom line is that suboptimal practices increase the chances of publishing inaccurate findings. This includes findings that are simply false and therefore irreplicable, but also more subtle errors, such as overclaiming, overgeneralizing, overestimating the power and pervasiveness of phenomena, overstating their societal significance, or suggesting that interventions be designed around findings with feet of clay. The extent to which these practices have produced invalid or overstated conclusions (Path i) is unclear, in part, because psychological scientists do not agree on what constitutes successful versus failed replications (compare Gilbert, King, Pettigrew, & Wilson, 2016 to Open Science Collaboration, 2015). Nonetheless, the mounting number of close pre-registered replications that find weak or no evidence of the original phenomenon muddies the credibility of the entire field (e.g., Finnigan & Corker, 2016; Yap et al., 2017).^2^

### The problem of canonization

**Figure 3:**
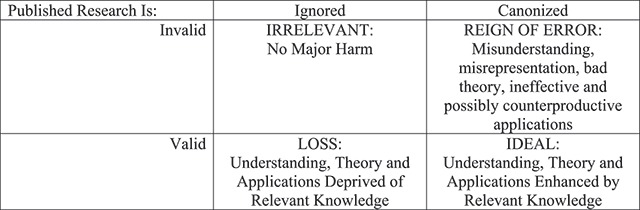
The Importance of Canonization.

Box 8, however includes not just the *publication* of erroneous findings, but their *canonization* and *dissemination*. Despite having received scant attention in the science reform movement to date, suboptimal practices in the canonization of findings can be viewed as the most important step in the model. Successfully getting *published* is necessary but hardly sufficient to make differences to either theory or applications unless the finding becomes part of a field’s received wisdom. Figure 3 captures what is at stake post-publication. If something invalid is published and ignored, its publication is not very important (the IRRELEVANT quadrant). However, if something invalid is published and canonized, it falls in the REIGN OF ERROR quadrant, whereby massive researcher effort, large amounts of grant dollars, and misguided interventions may come to rule the day. Widespread, but erroneous claims about the role of stress in producing ulcers (Blaser, 1996) is a clear example, and we might be in the midst of discovering that claims that stereotype threat causes gender gaps in math is another (Finnigan & Corker, 2016; Flore, Mulder, & Wicherts, 2019). If something valid is published but largely ignored, it falls in the LOSS quadrant because the information is there, it is knowable, but it is generally overlooked by theory and applications. Last, the IDEAL quadrant captures how optimal canonization works: valid findings are published and recognized, become canonized, and thereby enrich theory and enhance applications. In many ways, then, canonization matters more than publication.

What scientific standards warrant elevation of some finding to canonical status? The field currently has processes, but no articulated consensus or norms as to what should lead to high confidence in some conclusion. The process basically involves findings, claims, and conclusions making it into Annual Review and Handbook chapters, major textbooks, and the like. But what determines whether findings make it into those outlets of record? It is currently some unknown mix of popularity, prestige, having the right allies and supporters, compellingness of narrative and validity (Jussim et al., 2016; Merton, 1973). Only the latter has clear scientific merit. Even meta-analysis is no longer always considered a gold standard because studies using suboptimal practices may produce misleading meta-analytic results, and different approaches to meta-analysis sometimes yield different conclusions (van Elk et al., 2015). Psychology and other fields have delayed their own progress by leaping to canonical conclusions unsupported by robust evidence, and this sometimes takes many years to correct (Jussim, 2012; Loeb, 2014).

### Media misrepresentations, interventions, and societal value placed on psychology

This situation can be further exacerbated by media representations of flawed scientific claims (Box 9). Path j reflects media hyping bad research. Although journalists are fully capable of getting the science wrong on their own, two recent papers found that the primary source of inaccurate media representations is actually scientists themselves – who often present overstated or oversimplified descriptions of their findings to the media (Sumner et al., 2014, 2016).

Inasmuch as the media is often the main vehicle by which scientific claims come to be accepted as true by the public, one important downstream consequence is that many people may come to believe faulty claims. For example, many organizations have instituted diversity programs based, in part, around phenomena such as stereotype threat, microaggressions, and implicit biases, even though the scientific bases for considering these phenomena important contributors to social problems is dubious or, at best, controversial (e.g., Finnegan & Corker, 2016; Lilienfeld, 2017; Mitchell, 2018).

Path k therefore leads to Box 10, which represents failures of interventions to solve social problems. It should be apparent that interventions based on faulty or inadequately vetted scientific claims would mostly prove ineffective; and, if not, there is ample evidence that this is, in fact, the case (e.g., Blanton & Ikizer, 2018; Hanselman, Rozek, Grigg, & Borman, 2017; Rossi, 1987). Path l leads to the final box, 11, which constitutes a decline in value placed on social and behavioral science and scientists. As more and more failures characterize social interventions designed or inspired by flawed social science, a decline in public value and trust in science is both rational and likely (Edwards & Roy, 2017; Vazire, 2017).

One might reasonably wonder if focusing on flaws would similarly reduce public confidence in psychological science. Interestingly, some preliminary evidence suggests exactly the opposite. 194 college students taking sophomore/junior level psychology courses (courses in personality or methods) were asked to indicate how much they trusted psychology studies and how similar psychology is to natural science fields (Chopik, Bremner, Defever, & Keller, 2018). They were then asked the same questions *after* exposure to a one hour lecture on psychology’s replication crisis. Results showed that they did trust psychology studies less after the lecture – which seems entirely scientifically justified given the difficulty so many studies have replicating. In addition, however, those students also viewed psychology as more similar to natural science fields, a conclusion plausibly interpretable as seeing psychology as *more* scientific. Although we do not want to overinterpret the implications of this single modest study, it is at least some preliminary evidence suggesting that informing people about the flaws and weaknesses in psychological science can improve rather than harm its standing with the public.

## Proposed Solutions

**Figure 4:**
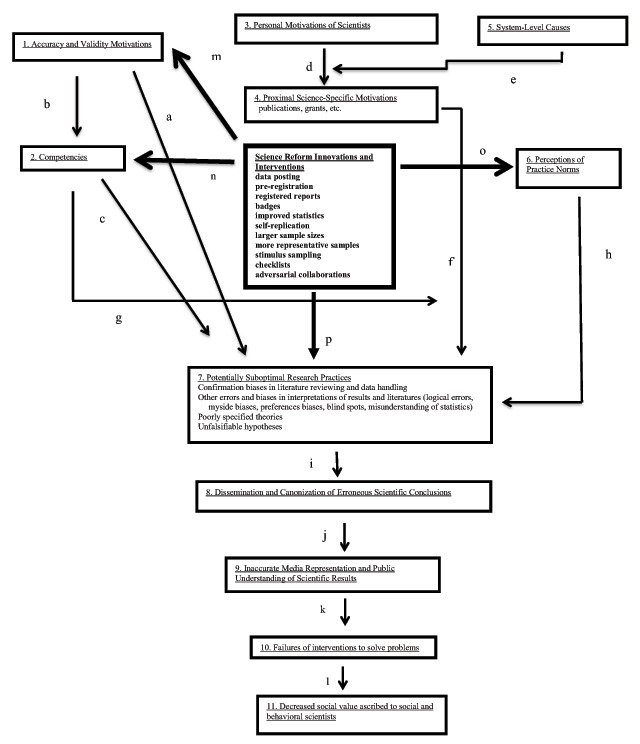
SPSPM-R, SPSPM With Reforms. This model is identical to the Social Psychological Model of Scientific Practices, with the following exceptions: The examples under each box header are not shown; a central box for science reforms has been added (in bold), as have several paths indicating how those reforms are predicted to influence scientific practices; Box 7 (Potentially Suboptimal Practices) only shows practices that are ***not*** expected to be affected by these reforms – all others are not shown. The new box and paths are shown with bold text and thicker boxes and arrows. Although there are no compelling reasons to predict that the major proposed reforms will alter the remaining suboptimal practices that are shown in Box 7, path p is included to permit the possibility that they may do so for reasons that are not yet well-understood. This model makes clear that most current reforms target statistics, methods, and practices, but not problems of logic or interpretation, nor the downstream consequences of inaccurate scientific conclusions.

Figure 4 is the SPMSP-Reform Model (SPSMP-R). It is identical to the main model in Figure 1 with one major addition: a central box has been added that lists existing or proposed interventions designed to improve psychological science (and often other fields as well). These include:

- Publicly and fully documenting and disseminating data and materials
- Pre-registration
- Registered reports
- Badges
- Use of improved statistical practices
- Increased sample sizes
- More representative samples
- Pressuring journals to be more open to publishing replications
- Development of forensic statistics for identifying literatures with greater or lesser evidentiary value
- Use of strong theories
- Including in papers statements about generalizability, falsification, etc.
- Practices to discourage theoretical and political confirmation biases (data blinding, checklists, adversarial collaborations, etc.).

In this paper, we do not review the history or rationale for these reforms, because they have been adequately described elsewhere (Brunner & Schimmack, 2017; Chambers, 2017; Fiedler, 2017; Funder et al., 2014; Giner-Sorolla, 2012; Heinrich et al., 2010; Nelson et al., 2018; Nosek et al., 2012; Odutayo et al., 2017; Spellman, 2015; Tybur & Navarette, 2018). Instead, we describe how the SPMSP-R offers a framework for understanding how – and testing whether – these and other initiatives reduce suboptimal practices and improve validity.

### Education

Scientists cannot possibly engage in optimal practices or avoid the worst suboptimal practices, unless they know how, when, and why to do so. In short, education is crucial to improving scientific practices. Nonetheless, education is not specifically shown in SPMSP-R because it has widespread and diffuse elements. Improved statistics and methods education can be viewed as a function of system-level causes (e.g., institutions and departments changing their methods and statistics requirements for students). At the same time, accuracy and validity motivations can cause researchers to seek out additional education via workshops, seminars, etc. And, in a broad sense of “formalized learning” (and not merely a narrow sense of “taking classes”), the science reform movement can be viewed as an education effort writ large, so that “education” is probably implicated in almost every box/variable shown in the SPSPM and SPSM-R. Thus, even though there is no specific variable shown in the SPSM-R, it is important to point out that exposure to improved scientific education, broadly construed, is a core component of efforts to improve the validity of psychological science.

### Transparency and Accountability

Many initiatives aim to increase transparency or accountability, and the SPMSP-R suggests that this might affect scientific processes in several ways: (1) by strengthening the effect of accuracy motives on research practices (path m); (2) increasing researchers’ competencies (path n); (3) and by changing social norms (path o). Empirical tests are needed assessing whether these changes will improve validity and replicability. One contribution of the SPMSP-I is to encourage scientists to embrace the supposed salutary effects of science reforms as hypotheses deserving investigation rather than as facts to be presumed.

Many reforms involve increasing transparency or accountability. Transparency refers to rendering research practices and results more available to other scientists. Accountability refers to rendering a scientist’s practices and findings subject to greater potential scrutiny by others. The two are synergistic, because transparency increases the opportunity for scientists to evaluate the methods and findings of their colleagues.

Disclosure reforms are intended to increase transparency. These include the 21-word solution (a statement declaring how sample size and data exclusions were decided and whether all conditions and measures are reported, Simmons, Nelson, & Simonsohn, 2012), as well as a variety of initiatives and support for researchers to post their materials and data in permanent online repositories available to other scientists. Pre-registration and, especially, registered reports (Chambers, 2017; Munafo et al., 2017) are intended to increase transparency by making more visible whether methods have been fully reported and which analyses were confirmatory vs. exploratory. By making publication contingent on the introduction, methods, and proposed analyses, but not the results, registered reports are also intended to mitigate many publication biases.

Transparency and accountability reforms are hypothesized to increase accuracy motivation for several reasons. First, accountability is most likely to elicit accuracy when four conditions are met (Lerner & Tetlock 1999): The audience: 1. Has views that are unknown; 2. is interested in accuracy; 3. is more interested in process than specific outcomes; and 4. is well-informed. This describes many scientists actively participating in science reform efforts (such as open data and pre-registration) quite well (e.g., Chambers, 2017; Funder et al., 2014; Nosek et al., 2012; Spellman, 2015). Furthermore, transparency renders one’s errors more visible to others, and we suspect few scientists want to be caught having made many errors.

Other reforms target accountability explicitly. Calls for larger sample sizes (Fraley & Vazire, 2014) hold researchers accountable for not capitalizing on the wider statistical variation inherent in small samples. Encouraging journals to publish replications (Funder et al., 2014) holds researchers accountable for not overselling initial findings – which rarely should be taken as “facts” pending extensive vetting and verification (preferably pre-registered) by independent researchers.

One mark of strong evidence is that it holds up even under intense skeptical scrutiny. Skepticism was once considered a core norm of science (Merton, 1973; Popper, 1959), a notion that may be undergoing a renaissance (Ioannidis, 2012). Post-publication peer review (Hunter, 2012) constitutes continued evaluation of the informativeness and proper interpretation of important studies after publication. Much currently takes place on social media, which is a source of controversy because lack of peer review-like controls means it sometimes involves heated rhetoric, personal insults, and hyperbole rather than reasonable engagements. Nonetheless, the Mertonian (1973) norm of skepticism seems to warrant some form of post-publication peer review.

Furthermore, new statistical tools can be used to evaluate the credibility and replicability of findings (Brunner & Schimmack, 2017). We call these “forensic techniques” because they evaluate the quality of research based on examination of the existing published product, usually its statistics. For example, p-curve analysis (Simonsohn, Nelson, & Simmons, 2014) determines whether the distribution of statistically significant p-values across a series of independent tests is consistent with a real or null effect. The Reproducibility Index uses all reported p-values in a paper to assess whether replication attempts are likely to succeed (www.r-index.org; Schimmack, 2014). These forensic techniques can be deployed to evaluate the credibility of whole research literatures, and have sometimes discovered that, despite there being a vast published literature attesting to some finding, the evidentiary value of that literature is exceedingly weak (Simmons & Simonsohn, 2017). That weak science can now be exposed in this way, therefore, constitutes a potentially powerful way to hold scientists accountable for producing credible findings.

In addition, many science reform efforts involve use of improved methods and statistics. This is captured by Path n, which means that many of these interventions require scientists to learn new competencies that are expected to improve the quality of their science (e.g., how to do pre-registration or registered reports, new statistical approaches, new methods, etc.). If these practices are, in fact, improvements, then by developing new competencies (Path n), suboptimal practices should be reduced (Path c).

Path o reflects the hypothesis that the recent wave of scientific reforms has some potential to create new norms. As pre-registration and replication become more common, as more journals *require* open data, as registered reports become more widely embraced, a tipping point may occur, whereby some suboptimal practices may atrophy and mostly disappear. If new standards become widely adopted, they may become normative, even if they are not strictly required. Whether people conform to new norms for informational reasons (they believe they are actually better) or normative reasons (everyone is doing it, so I have to, too), may not matter much with respect to practices. For example, why people use larger samples is probably less important than the mere act of using larger samples.

Path p captures the idea that many of these reforms and interventions might directly reduce suboptimal practices by virtue of *being* more optimal (i.e., without mediation by motivations, norms, etc.). Regardless of researchers’ skills or motivations, engaging in pre-registration increases the potential to distinguish between exploratory and confirmatory studies and analyses. Similarly, publishing registered reports may mitigate publication biases, regardless of anyone’s competencies or intentions. Adversarial collaborations can reduce reliance on unfalsifiable hypotheses by creating studies that use strong inference (Platt, 1964) – pitting opposing predictions generated by alternative theories against one another. These sorts of practices may directly reduce suboptimal ones.

## The Need for Empirical Research

Of course, just because an intervention is plausible, or widely-endorsed, does not mean that it is actually effective. We know of no scholar predicting that the science reform movement, or its innovations, will eliminate the very human tendencies to be self-serving and seek to game a system for personal gain. For example, if being able to declare that a study was pre-registered makes it easier to get the paper reporting that study published, it incentivizes pre-registration. Researchers will then be more likely to pre-register. Can pre-registration be gamed? It can: 1. Pre-register a study; 2. If the pre-registered analyses have problems or are judged to be insufficiently important, engage in exploratory analyses; 3. When writing up the paper, even if one does not intentionally engage in dubious practices, one can write up the paper in such a manner that, after referring to the pre-registration, does a poor job of clearly identifying which analyses were pre-registered. Therefore, without committing fraud, the paper can give the impression that exploratory analyses were confirmatory, as was routine in the bad old days pre-pre-registration (Kerr, 1998; Nelson et al., 2018). Similarly, one might prepare a pre-registration with eight key analyses, but write up a paper that only reports the two significant ones; in such a situation, the study was still, and truly, “pre-registered,” but the results would still appear more confirmatory or “beautiful” than they really are (for a real life example that comes close to this, see Wagenmakers & Gronau, 2017).

Furthermore, some have argued that many science reform efforts risk harming creativity (Baumeister, 2016); in fact, however, this may be the tip of an iceberg. Unintended (and currently unidentified) consequences may lurk as downsides of many science reform efforts. The extent to which reforms produce beneficial versus harmful effects are empirical questions. Thus, empirical research on the effectiveness of proposed solutions and interventions in psychology is needed. One contribution of the SPMSP-R, therefore, is to make predictions regarding when and how interventions are likely to influence scientists’ behavior to improve the rigor and quality of their research.

Although criticisms and innovations regarding scientific practices have always appeared in the journals, the post-2011 wave of reform has gotten much more traction than prior efforts (Gelman, 2016; Spellman, 2015). Nonetheless, research evaluating the effectiveness of interventions proposed or taken hold since 2011 is still in its infancy. Although a thorough review of every study evaluating the effectiveness of every proposed reform to statistics, methods, or practices is beyond the scope of the present paper, we do briefly review some of the evidence on the success of interventions post 2011.

Transparency and disclosure initiatives target norms and incentives. Badges are now offered by some journals for open data, open materials, and pre-registration. If the increased credibility that comes with earning badges increases visibility and citation metrics, they may become an incentive for (and lead to an increase in) open practices, which seems to be occurring (Kidwell et al., 2016). The SPMSP-R predicts that if transparency leads to greater accountability, then journals requiring more transparent practices (e.g., open data and materials) should be characterized by papers with fewer suboptimal practices. Evidence is beginning to come in suggesting this may be the case. For example, Schimmack (2017) found that social psychology journals, arguably at the core of much of psychology’s replication crisis, had dramatically improved scores on the replicability index.

Another hypothesis generated by the SPMSP-R is that pre-registration, and, especially, registered reports, should reduce publication biases (Path p). This should occur because articles are accepted primarily on the bases of evaluations of the importance of the research question and the quality of the methods – not the nature of the results. As a result, biases in favor of statistically significant findings, politically palatable findings, or consistency with famous theories by prestigious scientists should all be mitigated. Preliminary research suggests this, too, maybe happening. Whereas only 5–20% of conventionally published papers (without pre-registration) report findings that do not support the proposed hypotheses, nearly 2/3 of registered reports for replication studies do so, and more than half of all registered reports for novel studies do so (Warren, 2018). On the other hand, in biomedical research, pre-registration has had no effect on likelihood of a published report producing positive findings (Odutayo et al., 2017).

Another project tested hypotheses about the benefits of open practices (such as data posting) (Nuitjen, Borghuis, Veldkamp, Dominguez-Alvarez, van Assen, & Wicherts, 2017). One hypothesis was supported – more publications used open practices when journals required or incentivized them, supporting the idea that incentives and institutional practices matter directly (Path p). A second hypothesis, however, was not supported. Statistical reporting errors (e.g., consistency between a report t-value/degrees of freedom, and p-value) were not reduced. This fails to support the Path a hypothesis in the SPMSP-R: at least in this study, the increased accountability hypothesized to stem from making data available to other, potentially skeptical scientists, did not reduce errors. Whether this was because open data failed to increase feelings of accountability, or whether increased accountability did not reduce errors is currently unclear from this one study. Although this is only a single study, and more research is needed, it is possible that, to reduce such errors, interventions might need to target something else (e.g., Path c, perhaps statistical reporting errors are a result of competencies or carelessness rather than motivations).

Statistical reporting errors are only one type of quality issue, and it remains unknown whether open practices reduce other suboptimal practices. The “open practices produce fewer errors and biases” hypothesis of the SPMSP-R should be treated not as a single hypothesis, but as a family of related hypotheses. Those hypotheses could be tested with respect to individual studies and whole areas of research. For example, the SPMSP-R also predicts that open practices will reduce irreplicable findings and yield healthier-looking forensic analyses.

Many additional empirical questions can be generated on the basis of the SPMSP-R in the same way: Operationalize one or more variables in any box, treat the arrow from that box as a hypothesis predicting at least one of the (operationalized) outcomes in the next box. Regardless of what is ultimately *found*, a contribution of the SPMSP-R is that it can be used to generate empirically testable hypotheses about sources of optimal scientific practices. Because each box includes multiple constructs, each arrow represents multiple hypotheses.

The SPMSP-R also offers possible insight into how proposed interventions may fail to improve practices. Reforms may be gamed by researchers (Path f). For example, if claims to having conducted a pre-registered study increase the study’s publishability and impact, researchers will be incentivized to pre-register. However, except for registered reports, there are currently few standards for what a pre-registration document must say or how closely a published report must adhere to it. Researchers could propose many analyses in a pre-registration, and, in the published report, focus primarily on those that yielded statistically significant results, as has recently occurred (Wagenmakers & Gronau, 2017).

The SPMSP-R prediction that scientists are sometimes motivated to oversell their findings (Paths i and j) has been supported. Scientists often give their approval to overstated press releases that then produce inaccurate media coverage, as predicted by Path j (Sumner et al., 2016). An as-yet untested hypothesis is that if scientists’ personal motivations can lead to suboptimal practices then, when more is at stake, they should be more likely to use suboptimal practices. This can be used to identify conditions increasing suboptimal practices. Junior faculty may adopt suboptimal practices in their quest for tenure. Famous senior researchers may do so if they feel more ego-invested in the findings that made them famous. One important contribution of the SPSPM-R is that it can be easily used to generate hypotheses such as these for empirical investigation, thereby providing greater insight into which reforms are more effective at reducing suboptimal practices and increasing optimal practices.

## Limitations to Effectiveness of Science Reform Identified by the SPSPM

The SPSPM-R also identifies suboptimal practices that have not yet been targeted by major reform efforts – as such, there is no reason to expect changes in those practices. For example, statistical and methodological reforms do not target logical errors or systematic biases in interpretations or literature reviews (Jussim, 2012; Jussim et al., 2016). Nor do they address how findings become canonized or disseminated to the mass public. Thus, the SPMSP-R suggests that there is little reason to expect most currently proposed reforms to eliminate unjustified conclusions reached by logical errors, selective reviews of the literature, poorly specified theories, failure to consider alternative explanations, etc. Similarly, existing reforms do not discourage researchers from overselling results in public dissemination – and this can be expected to continue apace with existing downstream consequences until targeted reforms are instituted.

## Conclusion

The SPSPM provides a framework for summarizing and elaborating many ideas of the science reform effort, and for understanding the social psychology underlying when and why psychological scientists will adopt particular scientific practices. It constitutes a roadmap for identifying sources of suboptimal practices and key pressure points for interventions, and it highlights means of testing the effectiveness of those interventions. We concur with Munafò and colleagues (2017, p. 7) that “… offering a solution to a problem does not guarantee its effectiveness … Some solutions may be ineffective or even harmful to the efficiency and reliability of science, even if conceptually they appear sensible”. We join their call for increased investment in meta-science to provide evidence-based reforms to scientific practices.

The SPSPM, of course, has its own limitations. Its unique strength is primarily in bringing together many different avenues of scholarship in science reform; it is not a proposal for some bold new innovation intended to solve some particular problem. It is primarily a heuristic model providing a broad overview rather than a model that makes specific quantitative predictions. As result, the SPSPM is also limited in that it does not identify every single idea, suggestion, or recommendation for improving psychological science. For example, there is a burgeoning literature on limitations to meta-analysis, which some consider so severe as (Vosgerau, Simonsohn, Nelson, & Simmons, 2018) to warrant simply not using it, whereas others recognize limitations but recommend improving rather than abandoning it (Corker, 2019). One limitation of the SPSPM is that it does not do a deep dive into the specifics underlying such technical debates.

Instead, the SPSPM is intended to provide a bigger picture view of suboptimal practices and the reforms proposed to improve them. It synthesizes ideas that have been developed in the science reform movement into a single over-arching model (and improved meta-analysis would certainly fit into the “improved statistics” section of the SPSPM-R). It constitutes a framework for understanding sources of suboptimal practices, integrating some of the preliminary empirical research on these issues. Furthermore, it can be used to generate empirically testable hypotheses about how psychological science goes wrong and how to increase its chances of going right. As such, we hope that many of those who are not at the vanguard of the science reform movement (and maybe even a few that are) will find it useful.

## Footnotes

In most cases, the mirror image optimal practice is obvious (e.g., replace small samples with larger ones when possible) or is a topic of debate (whereas filedrawering is a problem, it is not obvious that publishing every study ever conducted is a solution).

See curatescience.org for a large list of replication attempts, many of which fail or produce considerably smaller effect sizes compared to the original studies.
